# Inhabiting the host: ectoparasites and Vector-Borne pathogens in Phyllostomidae bats within urban forest fragments

**DOI:** 10.1007/s11259-026-11341-x

**Published:** 2026-06-20

**Authors:** Nicolas Colácio, Yasmin C. Silva, Lucas B. S. Oliveira, Pedro G. S. Andrade, Leonardo P. Alcântara, Luiz G. M. Alves, Lis M. C. Vieira, Bruna H. Campos, Gabriel F. Diório, Brenda K. Gomes-Almeida, Marcelo P. N. Carvalho, Érika M. Braga, Júlia A. G. Silveira

**Affiliations:** 1 Laboratório de Protozoologia Veterinária (PROTOVET), Departamento de Medicina Veterinária Preventiva, Escola de Veterinária da Universidade Federal de Minas Gerais, Belo Horizonte, Minas Gerais Brazil; 2 Laboratório de Malária e Genômica de Parasitos, Instituto de Ciências Biológicas da, Universidade Federal de Minas Gerais, Belo Horizonte, Minas Gerais Brazil; 3 Departamento de Clínica e Cirurgia Veterinárias, Escola de Veterinária da Universidade Federal de Minas Gerais, Belo Horizonte, Minas Gerais Brazil; 4 Departamento de Medicina Veterinária Preventiva, Escola de Veterinária da Universidade Federal de Minas Gerais, Belo Horizonte, Minas Gerais Brazil; 5 Departamento de Zoologia, Instituto de Ciências Biológicas da Universidade Federal de Minas Gerais, Belo Horizonte, Minas Gerais Brazil

**Keywords:** Hemoparasites, Streblidae, Mesostigmata, *Ehrlichia* sp., Hemoplasma, *Leishmania* sp.

## Abstract

While bats are known to harbor various viruses and bacteria, their role as hosts for hemoparasites has not been extensively studied. This research investigated the presence of ectoparasites and vector-borne pathogens—including Anaplasmataceae agents, hemoplasmas, apicomplexans, and kinetoplastids—in 74 Phyllostomidae bats sampled from an urban forest fragment in southeastern Brazil. Overall, 31.1% of the bats were infested with ectoparasites: 82.6% carried only Streblidae bat flies, 13.0% had only mites, and 4.4% were co-infested with both parasites. No hemoparasites were observed in Giemsa-stained preparations. Furthermore, all samples tested negative for PCR assays targeting the orders Haemosporida and Piroplasmida, as well as for *Anaplasma* sp., *Neorickettsia* sp., *Trypanosoma cruzi*, and *Trypanosoma evansi*. Molecular analyses revealed that 1.4% of the bats were positive for *Ehrlichia* sp., 20.3% for hemotropic *Mycoplasma* spp., and 1.4% for *Leishmania infantum*. Coinfections were found in 1.4% of the positive samples. Sequence analyses revealed that the detected *Ehrlichia* sp. is closely related to *Ehrlichia canis*, and that the *Mycoplasma* sp. shares high sequence identity with bat-associated hemoplasmas. These findings document the presence of ectoparasites and hemopathogens in the sampled Phyllostomidae bats, indicating that these infectious agents circulate within urban forest fragments. While the specific role of these bats in sustaining the pathogens’ life cycles remains to be fully elucidated, these initial findings underscore the need for expanded eco-epidemiological studies. This highlights the crucial need to study these pathogens in bat populations, underscoring their ecological significance and the potential implications for public health and biodiversity conservation (e.g. zoonotic spillover and population stress).

**Supplementary Information:**

The online version contains supplementary material available at https://doi.org/10.1007/s11259-026-11341-x.

## Introduction

Bats (Mammalia: Chiroptera) comprise the second largest order of mammals worldwide (Hao et al. 2023). Due to their gregarious behavior, long lifespan, and unique ecological traits, bats are recognized as important reservoirs for a wide range of pathogenic agents (Dutheil et al. 2021). They also serve as hosts for a wide range of hematophagous ectoparasites, such as batflies and mites, many of which have been identified as vectors of bacterial and protozoan pathogens (Szentiványi et al. 2023).

Vector-borne diseases arise from a diverse array of pathogens, many of which have zoonotic potential and circulate among wildlife, livestock, and companion animals, forming complex epidemiological networks that pose substantial public health risks (Dantas-Torres and Otranto 2022). Within these networks, bacteria of the family Anaplasmataceae and hemotropic *Mycoplasma* species are of particular interest because they frequently co-circulate in wildlife and share overlapping transmission routes (Perles et al. 2023). Gram-negative bacteria of the family Anaplasmataceae, including *Anaplasma*, *Ehrlichia*, and *Neorickettsia*, are considered emerging agents of human diseases and hold great significance in Veterinary Medicine due to their substantial economic impact and effects on the health of domestic animals (Santos et al. 2017; Madison-Antenucci et al. 2020). Additionally, hemotropic *Mycoplasma* infects a wide range of mammals, potentially causing hemolytic anemia (Millán et al. 2015).

Among protozoan parasites, the orders Piroplasmida and Haemosporida have a significant impact on animal health, by driving substantial economic losses in livestock and compromising the fitness and survival of vulnerable wildlife populations through severe hemolytic diseases (Jalovecka et al. 2019; Pacheco and Escalante 2023). Finally, the family Trypanosomatidae, which includes *Leishmania* and *Trypanosoma*, comprises zoonotic agents responsible for diseases such as leishmaniasis (Camargo and Langoni 2006).

While bats are well-known reservoirs of emerging viral diseases (Han et al. 2015), they are increasingly recognized as key wildlife hosts within complex epidemiological networks of vector-borne pathogens (Alcantara et al. 2023). Therefore, this study aimed to investigate the occurrence of hemopathogens and ectoparasites in phyllostomid bats, contributing to a better understanding of parasite ecology and the potential role of bats as reservoirs in urban environments. These specific pathogens were selected because they share transmission networks and represent significant emerging threats to veterinary and public health at the urban-wildlife interface.

## Materials and methods

### Ethical statement

The study has received approval from the Ethics Committee on Animal Use (CEUA) at Universidade Federal de Minas Gerais (UFMG) under protocol number 172/2022. It is licensed and authorized by the Chico Mendes Institute for Biodiversity Conservation (ICMBio) through the Biodiversity Information and Authorization System (SISBIO) (83726-1), and registered in the National System for the Management of Genetic Heritage and Associated Traditional Knowledge (SISGEN) under the code A39E570.

### Study area

**Fig. 1:**
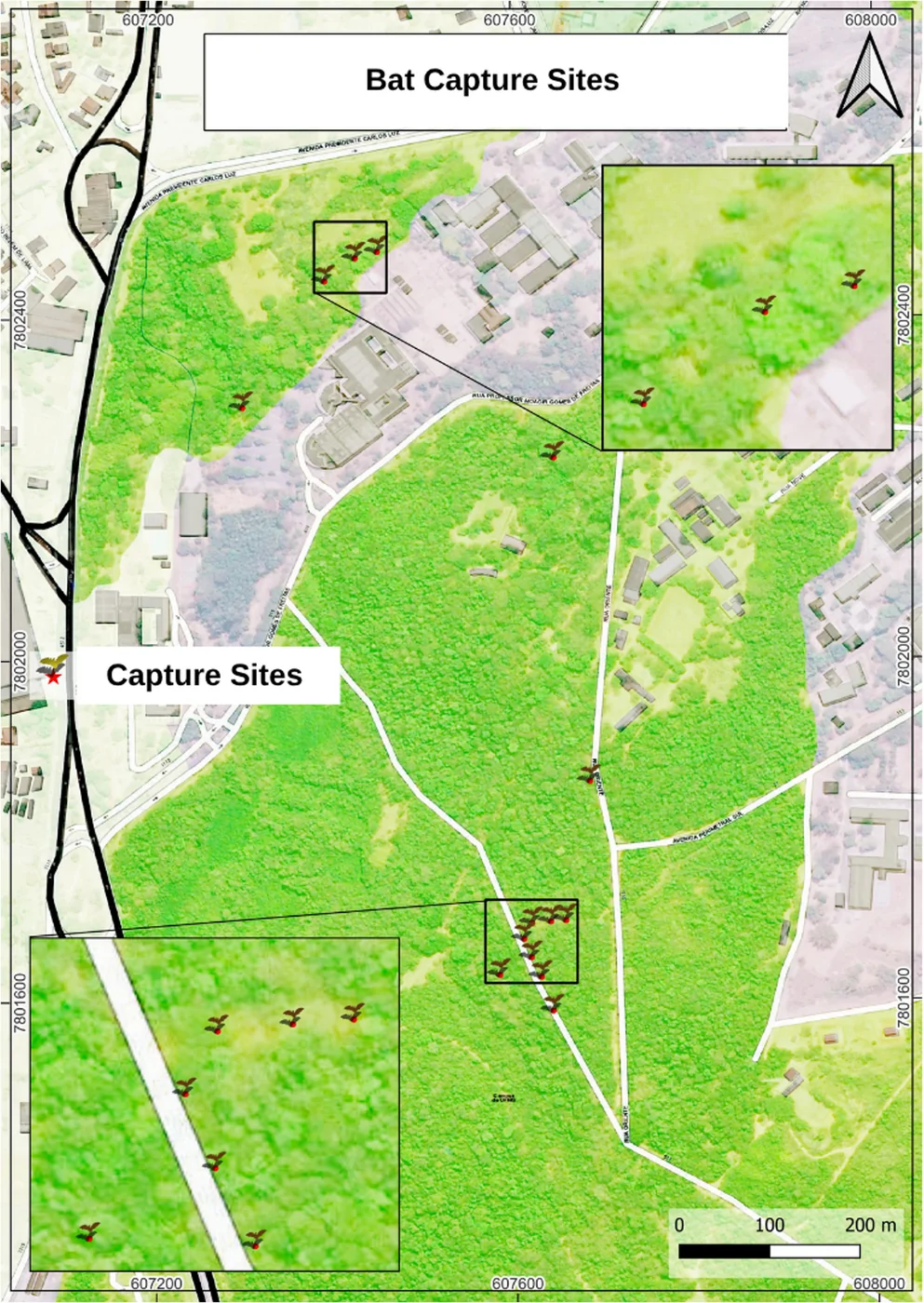
Spatial distribution of bat capture sites within the urban forest fragment of the Ecological Station (EECO), Belo Horizonte, Minas Gerais, Brazil. Red bat icons represent the specific geographic locations of mist net deployments. The magnified insets provide detailed views of clusters with a higher density of sampling points. Designed by Victor de Souza Rezende

The UFMG Ecological Station (EECO) is an urban environmental conservation unit situated on the Pampulha campus of the University in Belo Horizonte, Minas Gerais, Brazil. It covers an area of 114 hectares and encompasses 13 mapped biotopes, which host a rich diversity of species from both the Atlantic Forest and Cerrado biomes. The station is home to nine orders of mammals, 220 bird species, as well as reptiles, amphibians, and invertebrates, along with a wide variety of native and exotic plant species (Neves 2002). Additionally, EECO is located in close proximity to the university’s veterinary hospital, an area characterized by intense human activity and frequent movement of domestic animals (Fig. 1).

### Sampling information and microscopy

From October 2022 to December 2023, monthly capture campaigns were conducted at EECO. On each sampling night, two mist nets were opened at 18:00, kept open for four hours, and checked at 30-minute intervals. The bats were gently removed from the nets and transferred into individual bags. Measurements were taken to identify the bats to genus and/or species level using different identification keys (Reis et al. 2007; Díaz et al. 2021). The bats were examined for ectoparasites, which were collected and preserved in 70% ethanol. Subsequently, whole blood was collected by venipuncture of the cephalic vein, which was used to prepare two blood smear slides in the field. At the same time, the remaining sample was stored on untreated qualitative filter paper (80 g/m², 3 μm porosity; Biocentrix, Brazil) for subsequent molecular analyses. Finally, the bats were banded and released at the exact location where they were captured.

The slides were fixed with 100% methanol for 3 min and then stained with 10% Giemsa (pH = 7.2) for 70 min (Valkiunas et al. 2008). Giemsa-stained blood smears were examined for the presence of vector-borne pathogens, such as Anaplasmataceae, hemoplasmas, piroplasmids, haemosporidians, and kinetoplastids, using light microscopy with an Olympus CX31 microscope at × 1000 magnification with immersion oil.

### Ectoparasites

Mites specimens were mounted on microscope slides using Hoyer’s medium (Krantz and Walter 2009), identified to the species level, and deposited in the Acarological Collection of CCT-UFMG under the following numbers: UFMGAC-241,053 to UFMGAC-241,057. Morphology was observed and recorded using a camera attached to a Leica DM 750 light microscope, in accordance with the key of Radovsky (2010) and Gomes-Almeida et al. (2024). Morphological terminology of the flies follows Wenzel (1976) and Wenzel and Peterson (1987). Specimens were identified using the identification keys proposed by Wenzel (1976), supplemented by Guerrero (1995) for the *Trichobius* specimens. Flies were identified using a Leica M125 stereomicroscope at the Laboratory of Insect Systematics of ICB-UFMG. Slides were not mounted; instead, specimens were preserved in alcohol. The identified species were photographed with a Canon EOS R50 digital camera attached to a Leica M205 C stereomicroscope at the Laboratory of Arachnology of ICB-UFMG, using the EOS Utility 3 software. Image stacking was performed using Helicon Focus 8.2.18, and image editing was done in Adobe Photoshop 2019.

### DNA extraction and PCR amplification

DNA from bat blood was extracted using the “Wizard Genomic DNA Purification Kit” (Promega, Madison, WI, USA) according to the manufacturer’s recommendations. After the extraction, the quality and quantity of the samples were estimated using a NanoDrop (Epoch Microplate Spectrophotometer, Biotek^®^). The extracted DNA samples were stored at − 20 °C until they were subjected to PCR amplification. A fragment of the mammalian glyceraldehyde-3-phosphate dehydrogenase (*gapdh*) gene was used to verify the quality of the extracts (Birkenheuer et al. 2003).

Blood DNA samples from mammals that tested positive for the *gapdh* gene were subjected to PCR assays for *Anaplasma* sp., *Ehrlichia* sp., *Neorickettsia* sp., hemotropic *Mycoplasma*, piroplasmids, haemosporidians, and kinetoplastids. All agents, target genes, primer sequences, amplicon sizes (bp) and references are shown in Online Resource 1.

For the PCRs, positive DNA controls were obtained from various sources. These included *Babesia bovis* from an experimentally infected calf (strain BbovMG); *Ehrlichia canis*,* Anaplasma phagocytophilum*, and hemotropic *Mycoplasma* spp. from monkeys and dogs with confirmed sequences (Silveira et al. 2015; Castillo et al. 2024); *Plasmodium falciparum* from a blood culture (strain W2); *Trypansoma cruzi*, *T. evansi*, and *Leishmania* sp. (strain MCAN/BR/2002/BH400) from experimentally infected mice. Ultrapure sterile water (Life Technologies^®^, Carlsbad, CA, USA) was used as a negative control in all PCR assays.

PCR amplicons were separated by electrophoresis on 2% agarose gels (40 min, 100 V), stained with GelRedTM (Biotium, Hayward, CA, USA), and visualized under ultraviolet light.

### Restriction Fragment length polymorphism (RFLP) analysis—PCR for *Leishmania* spp

To identify *Leishmania* spp., the ITS1 amplicon was digested using the restriction enzyme HaeIII (PCR-RFLP). Five microliters of each amplified product were digested with the HaeIII restriction enzyme (Biolabs, Inc., UK) according to the manufacturer’s instructions. The digested products were subjected to 6% polyacrylamide gel electrophoresis to verify the restriction patterns. The restriction patterns obtained were compared with those of World Health Organization (WHO) reference strains (Schönian et al. 2003).

### Sequencing and phylogenetic analyses

Prior to sequencing, the PCR products were purified using the BioSpin PCR Purification Kit (BioFlux), following the manufacturer’s instructions. Sequencing was performed by ACTGene using an automatic sequencer (ABI 3730xl DNA Analyzer Applied Biosystems TM) with POP7 polymer and BigDye v3.1 using the same oligonucleotide primers used in the assays. For sequencing analysis, the raw data, in the form of chromatograms generated by the automatic sequencer “Analyzer Applied Biosystems Analyzer,” were aligned, edited, and analyzed using the BioEdit program, version 7.0.5.3 (Hall 1999). The identity of each sequence was confirmed by comparison with sequences available in GenBank using BLAST software (Basic Local Alignment Search Tool, http://blast.ncbi.nlm.nih.gov/) (Altschul et al. 1990). After comparing the identities, the analyzed sequences were classified according to the degree of similarity with data already deposited in GenBank. All nucleic acid sequences revealed in this study have been deposited in the GenBank database.

Multiple sequence alignments were carried out using MUSCLE multiple sequence alignment software, which was implemented in MEGA11 with previously published sequences in GenBank. Moreover, representative sample sequences were also downloaded for use as an outgroup in the phylogenetic analysis. Phylogenetic trees were constructed using maximum-likelihood (ML) methods based on the results of alignment with MEGA11 (Kimura 1980; Tamura 1992; Tamura and Nei 1993; Tamura et al. 2021), with suitable models selected according to the smallest Bayesian information criterion (BIC) score. The reliability of the tree topology was tested using 1000 bootstrap replicates, as implemented in the program (Felsenstein 1985).

## Results

Although the sampling design was not restricted to a specific taxonomic group, all bats captured in the mist nets belonged exclusively to the Phyllostomidae family. A total of 74 bats were captured and morphologically identified. Among the specimens, 31 were classified as *Artibeus lituratus*, 10 as *Glossophaga soricina*, nine as *Artibeus planirostris*, eight as *Phyllostomus discolor*, four as *Sturnira lilium*, two as *Platyrrhinus lineatus*, and one as *Carollia perspicillata*. However, nine individuals could not be identified to the genus level due to the absence of key biometric data. Although 74 bats were sampled, the sex of three individuals was unfortunately not recorded due to logistical constraints during fieldwork, of which 52.2% (37/71) were female and 47.8% (34/71) were male. All 74 blood-derived DNA samples yielded positive results in the cPCR assay targeting the *gapdh* gene and were therefore included in subsequent molecular analyses.

### Occurrence of ectoparasites

Ectoparasites were detected on 31.1% (23/74) of the sampled bats. Bat flies were the most prevalent, occurring exclusively on 82.6% (19/23) of the infested individuals, followed by exclusive mite infestations on 13.0% (3/23), and co-infestation by both taxa on 4.4% (1/23). Taxonomic identification of the 43 collected bat flies revealed a diverse community comprising *Trichobius costalimai* (25 specimens), *Paratrichobius longicrus* (6), *Megistopoda proxima* (4), *Aspidoptera falcata* (2), *Megistopoda aranea* (2), *Trichobioides perspicillatus* (2), *Trichobius angulatus* (1), and *Trichobius dugesii* (1). Regarding mites, only five individuals were collected due to field difficulties, such as the need to release highly stressed bats prematurely. Among these, four were identified as *Periglischrus iheringi* and one as *Macronyssus meridionalis* (Table 1). Images of all ectoparasites are provided in Online Resource 2.

**Table 1:** Identification of bat species with some type of ectoparasitism

ID	Species	Streblidae bat flies	Mites
M1	*Artibeus planirostris*	*Megistopoda aranea*	-
M3	*Phyllostomus discolor*	*Trichobius costalimai*	-
M7	*	*Trichobioides perspicillatus*	-
M11	*	*Trichobius costalimai*	-
M15	*Phyllostomus discolor*	*Trichobius costalimai*	-
M16	*Artibeus planirostris*	*Megistopoda aranea*	-
M17	*Artibeus lituratus*	*Paratrichobius longicrus*	-
M18	*Artibeus lituratus*	*Paratrichobius longicrus*	-
M20	*Platyrrhinus lineatus*	*Trichobius angulatus*	-
M21	*Artibeus lituratus*	*Paratrichobius longicrus*	-
M29	*Phyllostomus discolor*	*Trichobius costalimai*	-
M36	*Phyllostomus discolor*	*Trichobius costalimai*	-
M40	*Artibeus lituratus*	*Megistopoda proxima* and *Aspidoptera falcata*	-
M47	*Phyllostomus discolor*	*Trichobius costalimai*	-
M48	*Glossophaga soricina*	*Trichobius dugesii*	-
M50	*Artibeus lituratus*	*Paratrichobius longicrus*	-
M70	*Sturnira lilium*	*Paratrichobius longicrus*	-
M71	*Artibeus lituratus*	*Megistopoda proxima* and *Aspidoptera falcata*	-
M73	*Artibeus lituratus*	-	*Periglischrus iheringi*
M74	*Phyllostomus discolor*	*Trichobius costalimai*	-
M76	*Artibeus lituratus*	-	*Periglischrus iheringi*
M83	*Phyllostomus discolor*	*Trichobius costalimai*	*Periglischrus iheringi*
M88	*Artibeus lituratus*	-	*Macronyssus meridionalis*

*The identification of the bat species was not possible

### Blood smear analysis

No structure suggestive of vector-borne pathogens were found in blood smears from the 74 sampled bat hosts.

### Occurrence of anaplasmataceae agents

Out of 74 free-living bats, one *Artibeus lituratus* (1.4%) tested positive for *Ehrlichia* sp. based on the 16 S rRNA gene. That sample was subjected to another PCR reaction based on the 23 S–5 S ITS gene. However, the sample was negative in that reaction. The sequenced amplicon obtained from the 16 S rRNA gene (GenBank Accession Number: PQ576760) showed 99.32% of similarity with an isolate of *Ehrlichia canis* from Spain (GenBank Accession Number: KC479022). Phylogenetic analysis is provided in Online Resource 2. No bat tested positive for *Anaplasma* sp. or *Neorickettsia* sp.

### Occurrence of hemotropic *Mycoplasma*

Hemotropic *Mycoplasma* sp. was detected in 20.3% (15/74) using the 16 S rRNA gene cPCR assay. These samples were also subjected to the 23 S rRNA gene protocol, of which only 1.4% (1/74) were positive. To ensure high-quality sequencing results, four positive samples from the 16 S rRNA gene and one from the 23 S rRNA gene were selected based on the strong intensity of their amplicons observed during agarose gel electrophoresis. The 16 S rRNA sequences (GenBank Accession Numbers: PQ619014–PQ619017) showed 98.10% to 99.47% similarity to hemoplasmas previously identified in bats from Brazil and Belize (GenBank Accession Numbers: MZ048307; PQ764792; MH245136). The 23 S rRNA sequence (GenBank Accession Number: PV429979) displayed 98.65% similarity to a hemoplasma previously detected in *Artibeus jamaicensis* from Belize (GenBank Accession Number: OQ518935). Phylogenetic analyses are provided in Online Resource 2.

### Occurrence of protozoans

In the nPCR analysis for protozoans of the Trypanosomatidae family, 1.4% (1/74) were positive for *Leishmania* sp., which was confirmed as *Leishmania infantum* by PCR-RFLP. However, no bats tested positive for *T. cruzi*, *T. evansi*, the Piroplasmida or the Haemosporida orders. The complete PCR results are summarized in Table 2.

**Table 2:** Number of positive bat species for vector-borne pathogens in EECO

Bat family	Bat species	Number of individuals	Number of positive for Ehrlichia sp.	Number of positive for hemotropic Mycoplasma sp. (16 S rRNA)	Number of positive for hemotropic Mycoplasma sp. (23 S rRNA)	Number of positive for Leishmania infantum
Phyllostomidae	*Artibeus lituratus*	31	1	4	1	-
	*Artibeus planirostris*	9	-	2	-	-
	*Carollia perspicillata*	1	-	1	-	-
	*Glossophaga soricina*	10	-	-	-	-
	*Sturnira lilium*	4	-	-	-	-
	*Platyrrhinus lineatus*	2	-	-	-	-
	*Phyllostomus discolor*	8	-	2	-	-
	Unidentified Phyllostomidae	9	-	6	-	1
Total		74	1	15	1	1

### Co-infections detected

In the analysis of vector-borne pathogen coinfections, 1.4% of the bats (1/74) were found to be coinfected with *Leishmania infantum* and hemotropic *Mycoplasma* sp.

## Discussion

Areas of increased contact between wildlife and human-associated species (e.g., urban forest fragments, ecological corridors, and agricultural interfaces) are hotspots for potential zoonotic spillover and pathogen host shifts. In this study, we detected natural infections by vector-borne pathogens in phyllostomid bats inhabiting an urban forest fragment, including *Leishmania infantum*, a zoonotic protozoan of major public health concern (Morea et al. 2023).

In this study, 31.1% of the analyzed bats were ectoparasitized, with the majority of infestations (86.9%) caused by Streblidae bat flies. These flies demonstrated high host-specificity, with rare exceptions such as multiple parasitism in *A. lituratus* and the accidental presence of *P. longicrus* on *S. lilium* due to habitat overlap (Bertola et al. 2005; Dolabela-Falcão et al. 2022). Conversely, mites infested only 17.4% of the infested bats. Although field constraints hindered a comprehensive diversity assessment, two mite species were identified: *P. iheringi*, which aligns with existing literature (Bassini-Silva et al. 2025)d *meridionalis*, an unprecedented finding on *A. lituratus* that suggests either a broader host range than previously known or a case of accidental parasitism. Although no ticks were found on the bats examined, the role of these ectoparasites in pathogen transmission cannot be ruled out. This is particularly relevant given that the feeding behavior of soft ticks, commonly associated with bats, coincides with periods of bat inactivity within roosts (Muñoz-Leal et al. 2016).

In this study, molecular analysis revealed *Ehrlichia* sp. in 1.4% of the sampled bats based on the 16 S rRNA gene. Although this prevalence is lower than some international records (Zabashta et al. 2019), the detected sequence exhibited high similarity to *E. canis*. This contrasts with previous Brazilian studies that primarily detected genotypes resembling *E. minasensis* (de Mello et al. 2024), underscoring the need for additional molecular markers to achieve better phylogenetic resolution. Furthermore, hemotropic *Mycoplasma* sp. was detected in 20.3% (15/74) of samples based on the 16 S rRNA gene protocol, whereas amplification of the 23 S rRNA gene was achieved in only 1.4% (1/74). This discrepancy was anticipated due to the low sensitivity of the 23 S rRNA gene cPCR assay (Mongruel et al. 2022). The sequences obtained in this study showed similarity with other hemoplasmas from Neotropical bats in Brazil and Belize. This data reinforces the codivergence of Neotropical bats and their bacterial pathogens alongside rare and phylogenetically constrained host shifts (Alcantara et al. 2023). This genetic proximity suggests a host-pathogen codivergence in New World bats, which appear genetically distinct from Old World strains that are more closely related to zoonotic agents (Millán et al. 2015).

Notably, *L. infantum* was detected in 1.4% of the samples. Given that *L. infantum* is the causative agent of visceral leishmaniasis, a severe zoonosis that poses a significant public health challenge in the endemic area of Belo Horizonte (de Arruda et al. 2019), this finding suggests that chiropterans are exposed to the pathogen’s transmission network. While this low prevalence does not support a role as active dispersers, their exposure highlights the overlap of wildlife and vector populations in fragmented or peri-urban environments (Kuzmin et al. 2011). Addressing knowledge gaps regarding the vector specificity and host competence of bats is crucial for a deeper understanding of their ecological role in the epidemiology of vector-borne diseases and for enhancing integrated surveillance strategies.

Furthermore, the study also revealed a 1.4% co-infection rate involving *Mycoplasma* sp. and *L. infantum*, a concurrent infection that can severely exacerbate adverse health outcomes in the host by inducing severe hemolytic anemia, progressive immunosuppression, and multi-organ impairment (Bergmann et al. 2017). Overall, these findings emphasize the role of bats as microhabitats and potential dispersers of vector-borne pathogens in fragmented, peri-urban environments. Finally, it is important to acknowledge the limitations of the current study. The absence of morphological identification for some bat specimens, due to the lack of biometric data collection during fieldwork, hindered the accurate establishment of host-parasite associations. Furthermore, the sampling method may introduce bias, as bird capture using mist nets is influenced by multiple factors, including the presence of acute diseases (Grabow et al. 2024).

Our study highlights the diversity of ectoparasites and vector-borne pathogens that infect and co-infect New World leaf-nosed bats in an urban forest fragment in Southeast Brazil, including zoonotic species. While these molecular findings confirm bat exposure to diverse vector-borne agents, they highlight the critical need to further investigate whether these mammals act merely as incidental hosts or true reservoirs in these urban ecosystems. Future research must assess these risks practically by evaluating the clinical and hematological parameters of infected bats, and by conducting vector competence assays to determine if local ectoparasites can effectively transmit these pathogens to humans and domestic animals moving through the university campus.

## Supplementary Information

Below is the link to the electronic supplementary material.

Supplementary Material 1

Supplementary Material 2

## Data Availability

No datasets were generated or analysed during the current study.
