# Supplementary material for: Inhabiting the host: ectoparasites and Vector-Borne pathogens in Phyllostomidae bats within urban forest fragments
Source: Vet Res Commun. 2026 Jun 20;50(5):407. doi: 10.1007/s11259-026-11341-x (PMC13283151; doi:10.1007/s11259-026-11341-x)
Supplement: Supplementary file 1 — Supplementary Material 1 [file 11259_2026_11341_MOESM1_ESM.docx]

**Supplementary Table 1.** Molecular assays used in the present study are categorized by the involved agent, type of PCR, target gene, primer sequences, amplicon size (bp), thermal cycling conditions, and references.

| **Agents** | **Aim/Primer** | **Molecular assay** | **Primers sequences** | **Fragment size (bp)** | **Thermal Cycling Conditions** | **Reference** |
| --- | --- | --- | --- | --- | --- | --- |
| ***Anaplasma* sp.**  (16S rRNA gene)  screening | 1ª reaction  gE3a  gE10R  2ª reaction  gE2  gE9f. | nPCR | 5′-CACATGCAAGTCGAACGGATTATTC-3′  5′-TTCCGTTAAGAAGGATCTAATCTCC′-3′  5′-GGCAGTATTAAAAGCAGCTCCAGG-3′  5′-AACGGATTATTCTTTATAGCTTGCT-3′ | 932  546 | 94◦C for 5min 40 cycles: 94◦C for 30s, 55◦C for 30s and 72◦C for 1min, 72◦C for 5min | Massung et al., 1998 |
| ***Ehrlichia* sp.**  (16S rRNA gene)  screening | 1ª reaction  NS16SCH1F  NS16SCH1R  2ª reaction  NS16SCH2F  NS16SCH2R | nPCR | 5′-ACGGACAATTGCTTATAGCCTT-3’  5′-ACAACTTTTATGGATTAGCTAAAT-3’  5′-GGGCACGTAGGTGGACTAG-3′  5′-CCTGTTAGGAGGGATACGAC-3′ | 1195  443 | 94◦C for 5min 30 cycles: 92◦C for 1min, 54◦C for 1 min and 72◦C for 2min, 72◦C for 8min | Kawahara et al., 2009 |
| ***Ehrlichia* sp.**  (23S-5S ITS region)  characterization | 1ª reaction  ITS2F  ITS2R  2ª reaction  ITS2iF  ITS2iR | nPCR | 5’-AGGATCTGACTCTAGTACGAG-3’  5’-CTCCCATGTCTTAAGACAAAG-3’  5’-ATACCTCTGGTGTACCAGTTG-3’  5’-TTAACTTCCGGGTTCGGAATG-3’ | *  300 | 94ºC for 2 min, 35 cycles: 94ºC for 30 s, 58ºC for 30 s and 72ºC for 1 min, 72ºC for 5 min | Rejmanek et al., 2012 |
| ***Neorickettsia* sp.** (16S rRNA gene)  screening | 1ª reaction  ER3  ER2  2ª reaction  ER3A  ER2A | nPCR | 5’-ATTTGAGAGTTTGATCCTGG-3’  5’-GTTTTAAATGCAGTTCTTGG-3’  5’-CTAGCGGTAGCGTTAAC-3’  5’-CACACCTAACTTACGGG-3’ | *  527 | 94°C for 5 min, 30 cycles: 94°C for 1 min, 60°C for 2 min, and 72°C for 1 min, 72°C for 7 min | Chae et al., 2003 |
| **Hemotropic *Mycoplasma* sp**.  (16S rRNA gene)  screening | HBTF  HBTR | cPCR | 5′-ATACGGCCCATATTCCTACG-3′  5′-TGCTCCACCACTTGTTCA-3′ | 618 | 94◦C for 10min, 40 cycles: 95◦C for 30s, 60◦C for 30s, 72◦C for 30s, 72◦C for 10min | Criado-Fornelio et al., 2003 |
| **Hemotropic *Mycoplasma* sp**.  (23S rRNA gene)  characterization | 23S_HAEMO_F  23S_HAEMO_R | cPCR | 5’-TGAGGGAAAGAGCCCAGAC-3’  5’-GGACAGAATTTACCTGACAAGG-3’ | 800 | 94 °C for 3 min, 35 cycles: 94 °C for 30 s, 54°C for 30 s, 72 °C for 1 min, and 72 °C for 10 min, | Mongruel et al., 2020 |
| **Piroplasmida**  (18S rRNA gene)  screening | 1ª reaction  RIB-19  RIB-20  2ª reaction  BabRumF  BabRumR | nPCR | 5′-CGGGATCCAACCTGGTTGATCCTGC-3′  5′-CCGAATTCCTTGTTACGACTTCTC-3′  5′-ACCTCACCAGGTCCAGACAG -3′  5′-GTACAAAGGGCAGGGACGTA-3′ | 1700  430 | 94◦C for 5min 30 cycles: 92◦C for 1min, 54◦C for 1 min and 72◦C for 2 min, 72◦C for 8 min | Zahler et al., 2000  Silveira et al., 2011 |
| **Haemosporida**  (*cytb* gene)  screening/characterization | 1ª reaction  DW2  DW4  2ª reaction  DW1  DW6 | nPCR | 5’-TAATGCCTAGACGTATTCCTGATTATCCAG-3’  5’-TGTTTGCTTGGGAGCTGTAATCATAATGTG-3  5’-TCAACAATGACTTTATTTGG-3’  5′-GGGAGCTGTAATCATAATGTG-3′ | *  1116 | 94◦C for 4min, 35 cycles: 94◦C for 1min, 55◦C for 15s, 72◦C for 1min and 15s, 68◦C for 10min | Perkins and Schall, 2002 |
| ***Trypanosoma evansi***  (*RoTat 1.2 VSG* gene)  screening/characterization | TVSGF  TVSGR | cPCR | 5’-GGGAATTCATGCAAACCAAGGCGCTCGTTGGCGT-3’  5’-CGGGAATTCCTTGATGTTGCTGGTCGCGATTTTGATC-3’ | 681 | 95◦C for 2 min, 35 cycles: 94◦C for 45 s, 60◦C for 1 min, and 72◦C for 2 min, and 72◦C for 10 min | Sengupta et al., 2012 |
| ***Leishmania* sp.**  (*ITS1* region)  screening/characterization | LITSR  L5.8S | cPCR | 5’-CTGGATCATTTTCCGATG-3’  5’-TGATACCACTTATCGCACTT-3’ | 300-350 | 95◦C for 2min, 37 cycles: 94◦C for 30s, 53◦C for 1min, 72◦C for 1min, 72◦C for 6min | El Tai et al., 2000 |
| ***Trypanosoma cruzi***  (*kDNA* gene)  screening/characterization | S35  S36 | cPCR | 5’-AAATAATGTACGGGKGAGATGCATGA-3’ 5’-GGTTCGATTGGGGTTGGTGTAATATA-3’ | 333 | 95◦C for 5min, 30 cycles: 95◦C for 1min, 60◦C for 1min, 72◦C for 1min, 72◦C for 5min | Vallejo et al., 1999 |

**References**

Chae, J. S., Kim, E. H., Kim, M. S., Kim, M. J., Cho, Y. H., & Park, B. K. (2003). Prevalence and sequence analyses of *Neorickettsia risticii*. *Ann. N. Y. Acad. Sci.*, 990, 248–256. https://doi.org/10.1111/j.1749-6632.2003.tb07372.x

Criado-Fornelio, A., Martinez-Marcos, A., Buling-Saraña, A., & Barba-Carretero, J. C. (2003). Presence of *Mycoplasma haemofelis*, *Mycoplasma haemominutum* and piroplasmids in cats from southern Europe: a molecular study. *Vet. Microbiol.*, 93(4), 307–317. https://doi.org/10.1016/s0378-1135(03)00044-0

El Tai, N. O., Osman, O. F., El Fari, M., Presber, W., & Schönian, G. (2000). Genetic heterogeneity of ribosomal internal transcribed spacer in clinical samples of *Leishmania donovani* spotted on filter paper as revealed by single-strand conformation polymorphisms and sequencing. *Trans. R. Soc. Trop. Med. Hyg.*, 94(5), 575-579. https://doi.org/10.1016/S0035-9203(00)90093-2

Kawahara, M., Tajima, T., Torii, H., Yabutani, M., Ishii, J., Harasawa, M., & Rikihisa, Y. (2009). *Ehrlichia chaffeensis* infection of sika deer, Japan. *Emerging Infect. Dis.*, 15(12), 1991. <https://doi.org/10.3201/eid1512.081667>

Massung, R. F., Slater, K., Owens, J. H., Nicholson, W. L., Mather, T. N., Solberg, V. B., & Olson, J. G. (1998). Nested PCR assay for detection of granulocytic ehrlichiae. *J. Clin. Microbiol.*, 36(4), 1090–1095. <https://doi.org/10.1128/JCM.36.4.1090-1095.1998>

Mongruel, A. C. B., Spanhol, V. C., Valente, J. D. M., Porto, P. P., Ogawa, L., Otomura, F. H., Marquez, E. S., André, M. R., Vieira, T. S. W. J., & Vieira, R. F. D. C. (2020). Survey of vector-borne and nematode parasites involved in the etiology of anemic syndrome in sheep from Southern Brazil. *Rev. Bras. Parasitol. Vet.,* 29(3), e007320. https://doi.org/10.1590/S1984-29612020062

Perkins, S. L., & Schall, J. J. (2002). A molecular phylogeny of malarial parasites recovered from cytochrome b gene sequences. *J. Parasitol.,* 88(5), 972–978. https://doi.org/10.1645/0022-3395(2002)088[0972:AMPOMP]2.0.CO;2

Rejmanek, D., Bradburd, G., & Foley, J. (2012). Molecular characterization reveals distinct genospecies of Anaplasma phagocytophilum from diverse North American hosts. *J. Med. Microbiol*., 61(Pt 2), 204–212. https://doi.org/10.1099/jmm.0.034702-0

Sengupta, P. P., Balumahendiran, M., Balamurugan, V., Rudramurthy, G. R., & Prabhudas, K. (2012). Expressed truncated N-terminal variable surface glycoprotein (VSG) of *Trypanosoma evansi* in *E. coli* exhibits immuno-reactivity. *Vet. Parasitol*., 187(1-2), 1–8. https://doi.org/10.1016/j.vetpar.2012.01.012

Silveira, J. A., Rabelo, E. M., & Ribeiro, M. F. (2011). Detection of *Theileria* and *Babesia* in brown brocket deer (*Mazama gouazoubira*) and marsh deer (*Blastocerus dichotomus*) in the State of Minas Gerais, Brazil. *Vet. Parasitol*., 177(1-2), 61–66. https://doi.org/10.1016/j.vetpar.2010.10.044

Vallejo, G. A., Guhl, F., Chiari, E., & Macedo, A. M. (1999). Species specific detection of *Trypanosoma cruzi* and *Trypanosoma rangeli* in vector and mammalian hosts by polymerase chain reaction amplification of kinetoplast minicircle DNA. *Acta Trop.*, 72(2), 203–212. https://doi.org/10.1016/s0001-706x(98)00085-0

Zahler, M., Rinder, H., Schein, E., & Gothe, R. (2000). Detection of a new pathogenic Babesia microti-like species in dogs. *Vet. Parasitol.*, 89(3), 241–248. https://doi.org/10.1016/s0304-4017(00)00202-8
