# Supplementary material for: Inhabiting the host: ectoparasites and Vector-Borne pathogens in Phyllostomidae bats within urban forest fragments
Source: Vet Res Commun. 2026 Jun 20;50(5):407. doi: 10.1007/s11259-026-11341-x (PMC13283151; doi:10.1007/s11259-026-11341-x)

**SFig. 1:** *Trichobius costalimai*, male. A - dorsal view. B - ventral view. C - thorax, dorsal view; large area without setae. D - thorax, ventral view; metasternal lobe (surrounded by the red dashed circle) present and connected to the metepimeron. Scale bars: A = 0.5 mm, B = 0.5 mm, C = 0.2 mm, D = 0.2 mm.


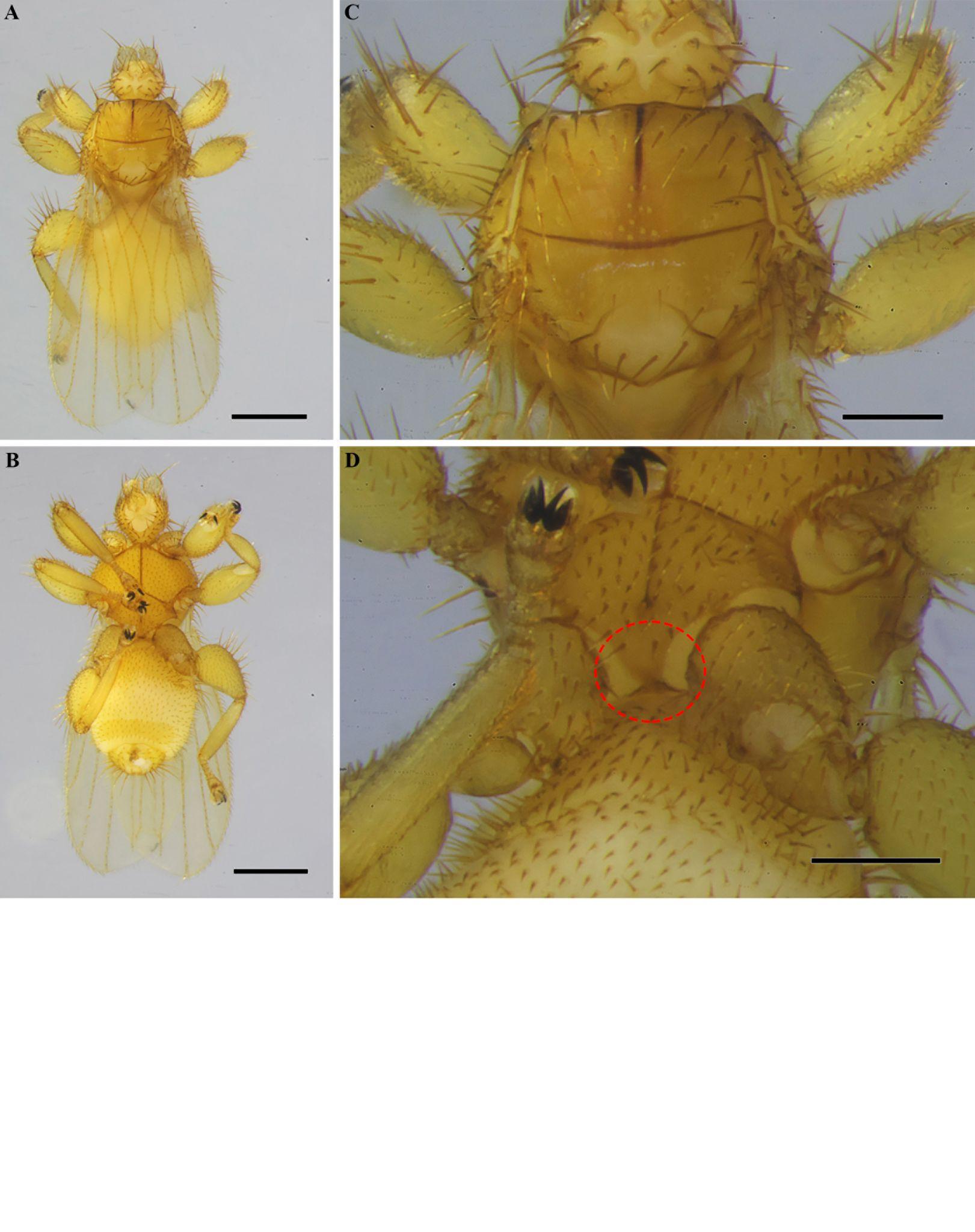


**SFig. 2:** *Paratrichobius longicrus*, male. A - dorsal view. B - ventral view. C - right fore leg, dorsal view; fore femur with one row of six spine-like setae (indicated by the red arrows). Scale bars: A = 1 mm, B = 1 mm, C = 0.2 mm.


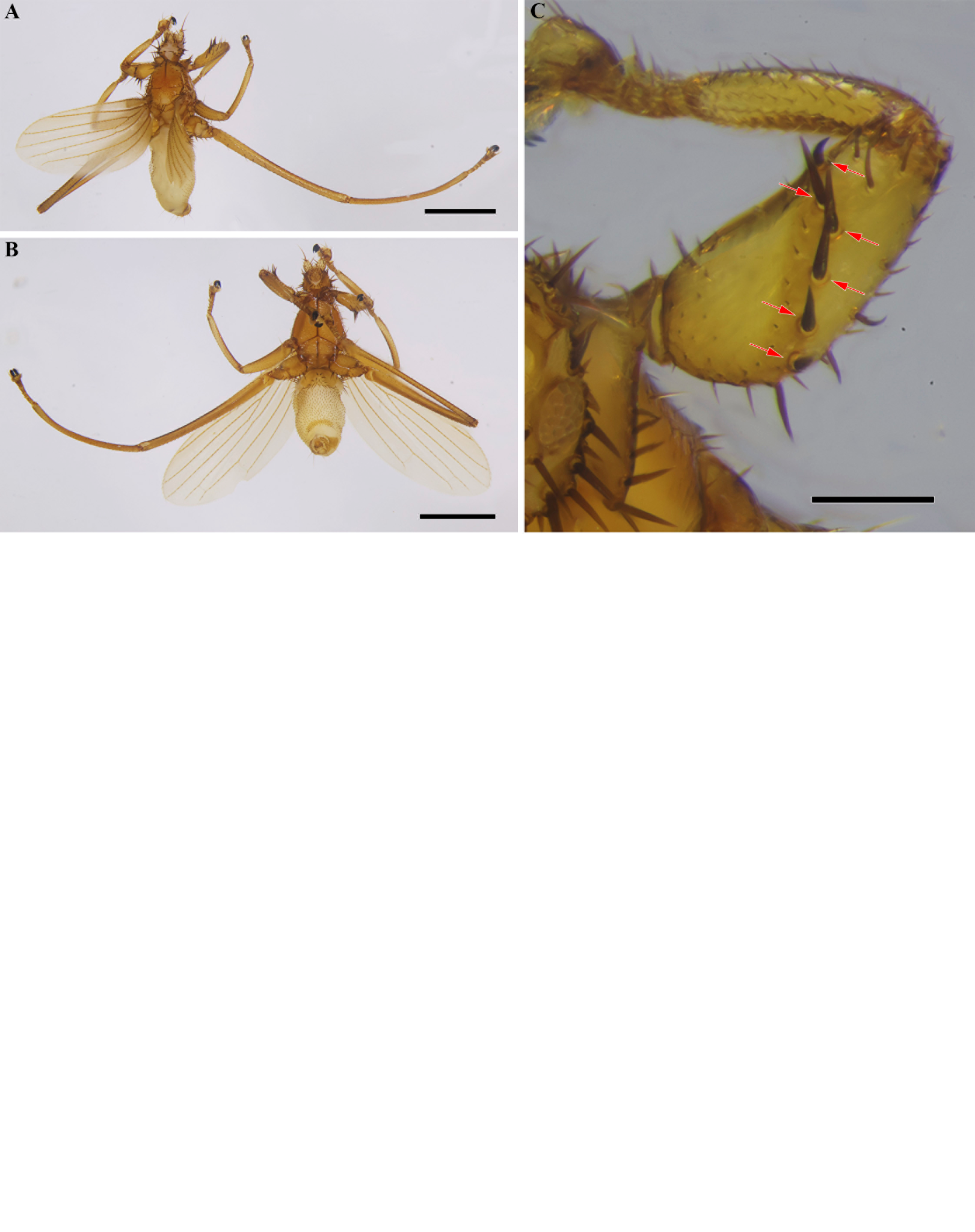


**SFig. 3.** *Megistopoda proxima*, male. A - dorsal view. B - ventral view. C - wings, left lateral view, reduced and narrow wings, with more than four longitudinal veins only (indicated by the red arrows). D - scutellum, dorsal view; four macrosetae. Scale bars: A = 1 mm, B = 1 mm, C = 0.2 mm, D = 0.2 mm.


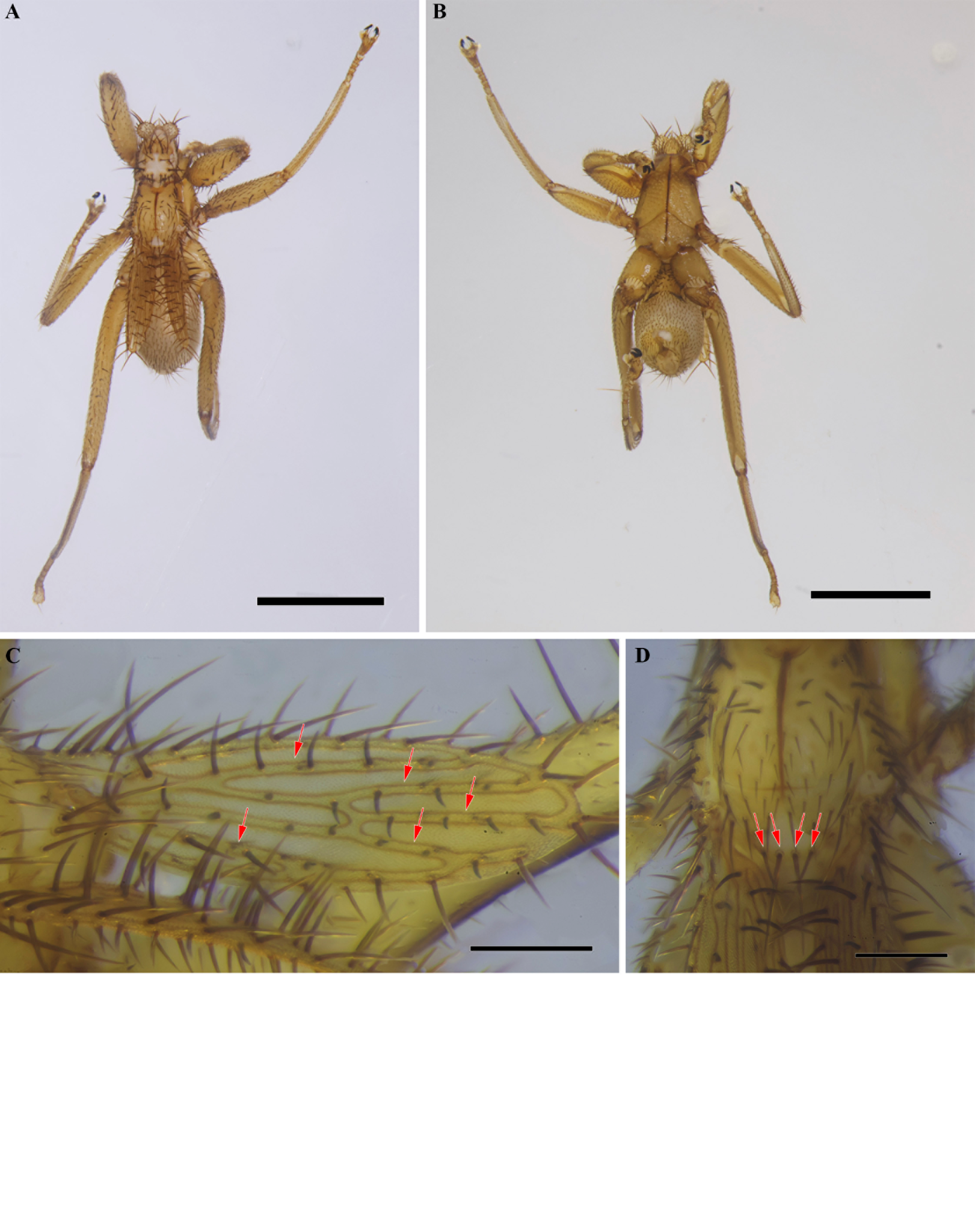


**SFig. 4:** *Aspidoptera falcata*, male. A - dorsal view. B - ventral view. C - thorax, dorsal view; longitudinal median suture (indicated by the red arrows) connected to transverse suture, forming an inverted “T”. D - thorax, left lateral view; mesepisternum, upper portion with three rows of longitudinal longer setae (surrounded by the red dashed circle). E - male abdomen, left lateral view; male gonopods (indicated by the red arrow) strongly bent, falciform. Scale bars: A = 0.5 mm, B = 0.5 mm, C = 0.2 mm, D = 0.2 mm, E = 0.2 mm.


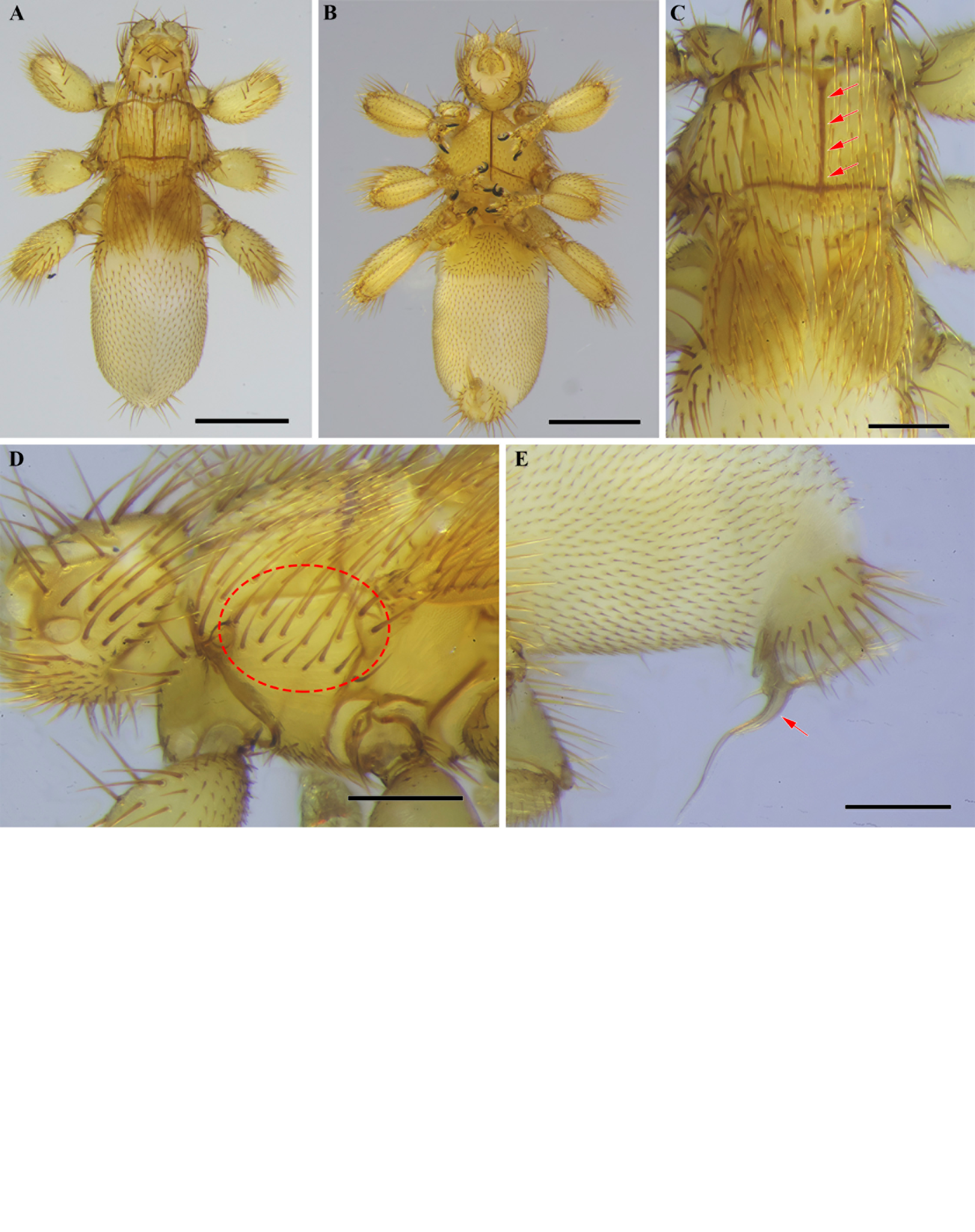


**SFig. 5:** *Megistopoda aranea*, female. A - dorsal view. B - ventral view. C - wings, left lateral view, reduced and very narrow wings, with four longitudinal veins only (indicated by the red arrows). D - scutellum, dorsal view; two macrosetae. Scale bars: A = 1 mm, B = 1 mm, C = 0.2 mm, D = 0.2 mm.


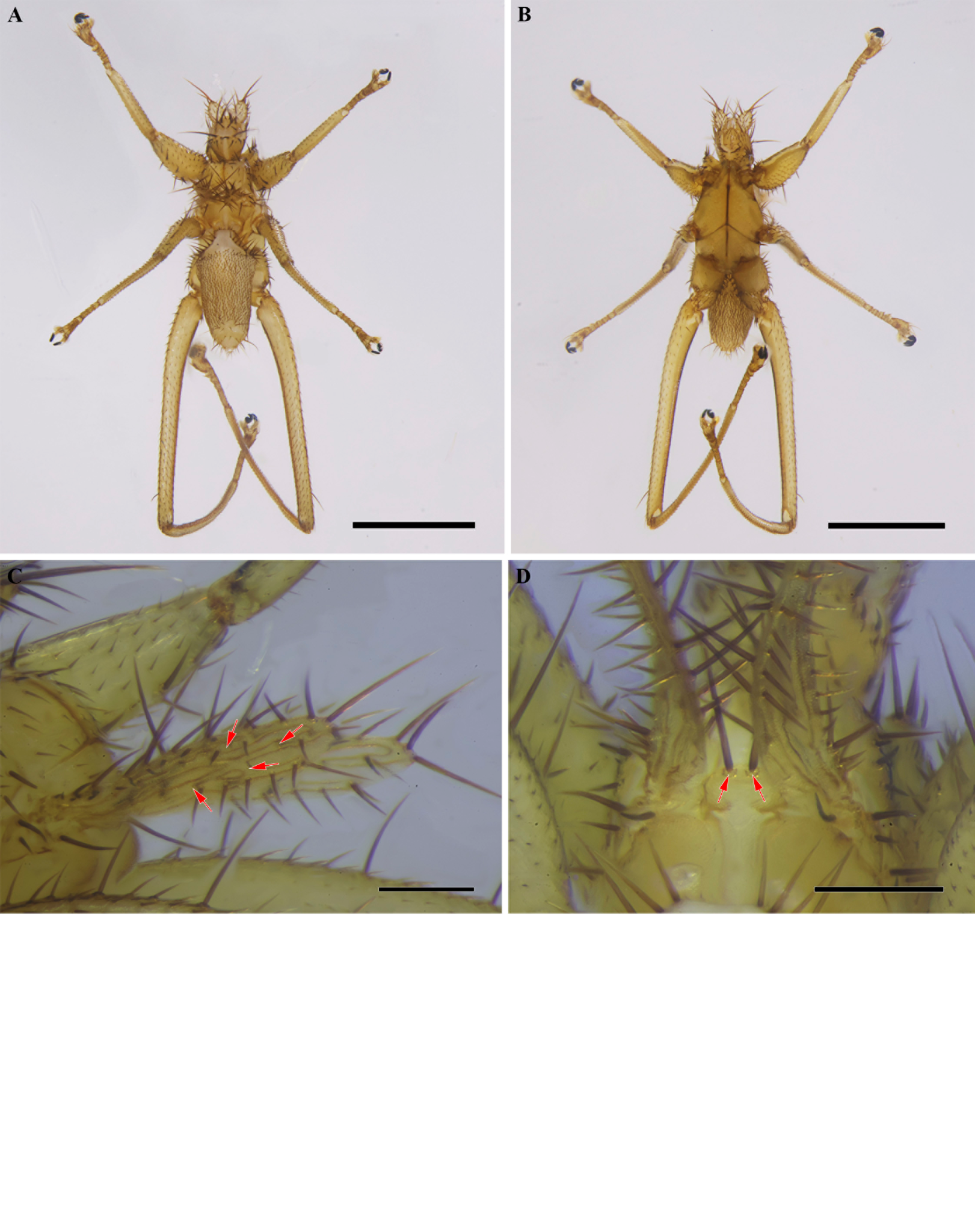


**SFig. 6.** *Trichobioides perspicillatus*, male. A - dorsal view. B - ventral view. C - head, anterior view; palpi (indicated by the red arrows) bare in the ventral surface. D - left wing, dorsal view; C (indicated by the red arrows) and R 1 (indicated by the green arrows) veins covered with strong macrosetae. Scale bars: A = 0.5 mm, B = 0.5 mm, C = 0.2 mm, D = 0.2 mm.


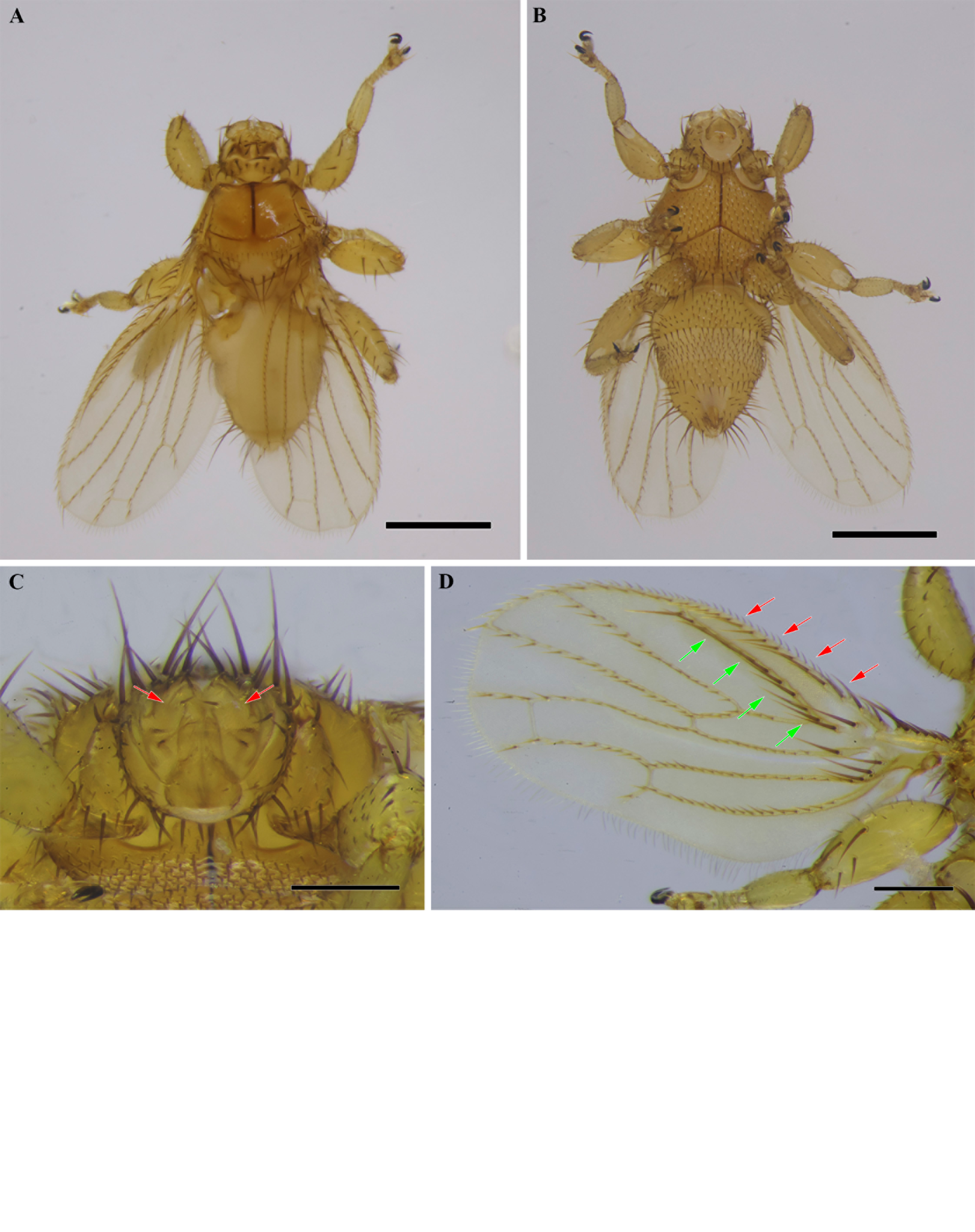


**SFig. 7.** *Trichobius angulatus*, male. A - dorsal view. B - ventral view. C - head, ventral view; palpi (indicated by the red dashed circle) with more than half its surface covered with setae. D - thorax, dorsal view; transverse suture (indicated by the red arrows) very angulate. E - thorax, ventral view; metasternal lobe (surrounded by the red dashed circle) present and not connected to the metepimeron. Scale bars: A = 0.5 mm, B = 0.5 mm, C = 0.2 mm, D = 0.2 mm, E = 0.2 mm.


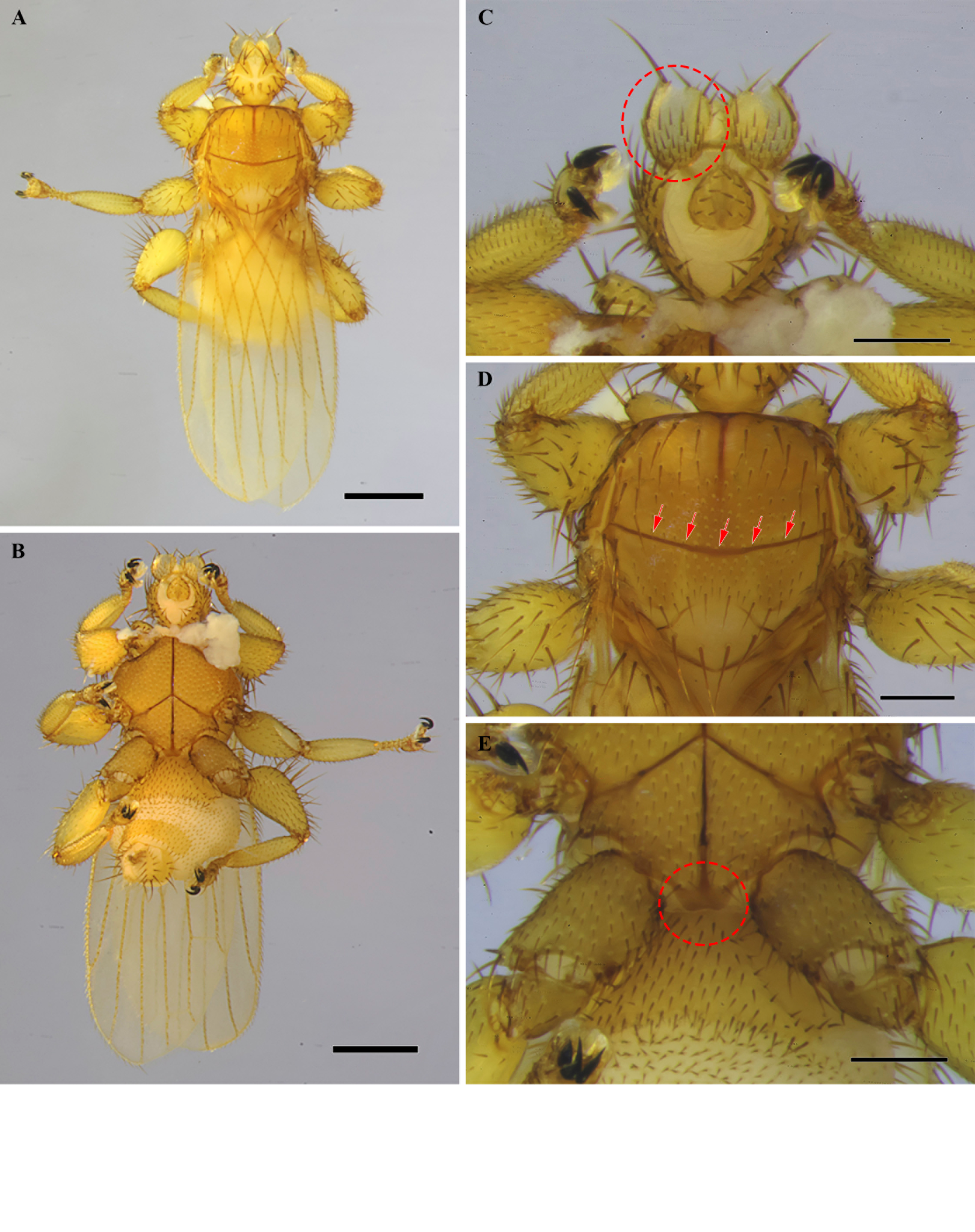


**SFig. 8.** *Trichobius dugesii*, female. A - dorsal view. B - ventral view. C - thorax, dorsal view; antescutellar row of setae with a mixture of longer setae (indicated by the red arrows) and shorter setae (indicated by the green arrows). D - thorax, ventral view; metasternal lobe (surrounded by the red dashed circle) present and not connected to the metepimeron. Scale bars: A = 0.5 mm, B = 0.5 mm, C = 0.2 mm, D = 0.2 mm.


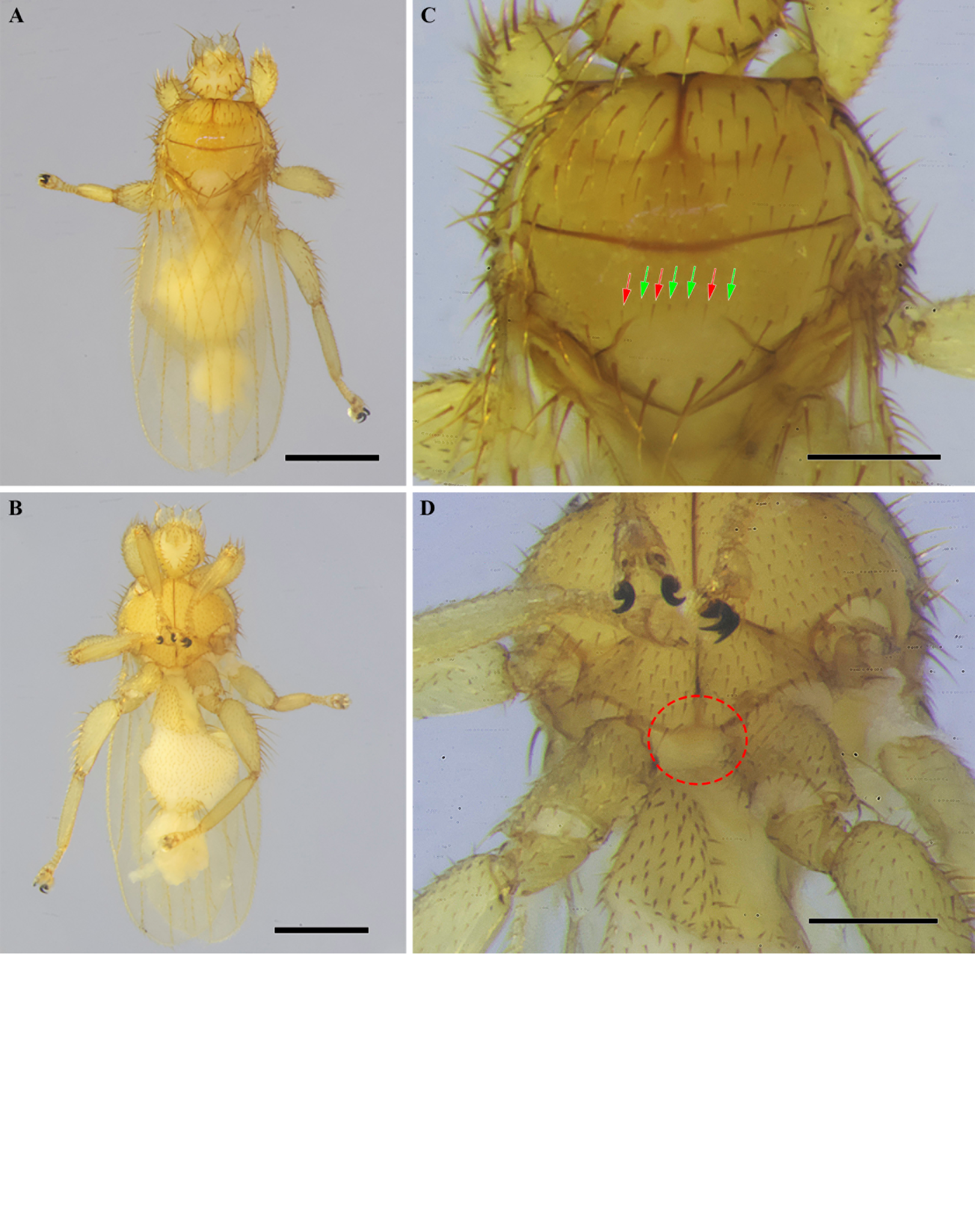


**SFig. 9.** *Periglischrus iheringi*, male.


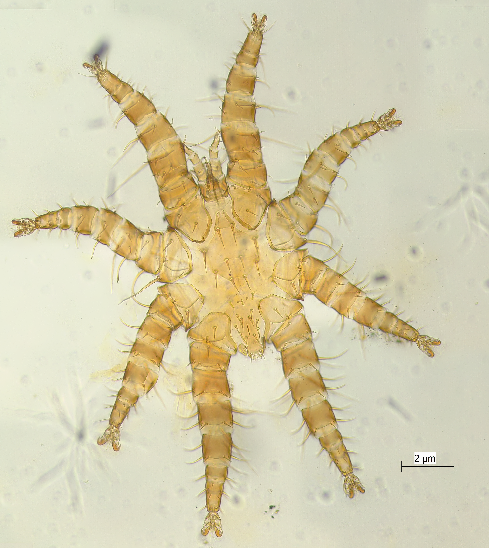


**SFig. 10.** *Macronyssus meridionalis*, female.


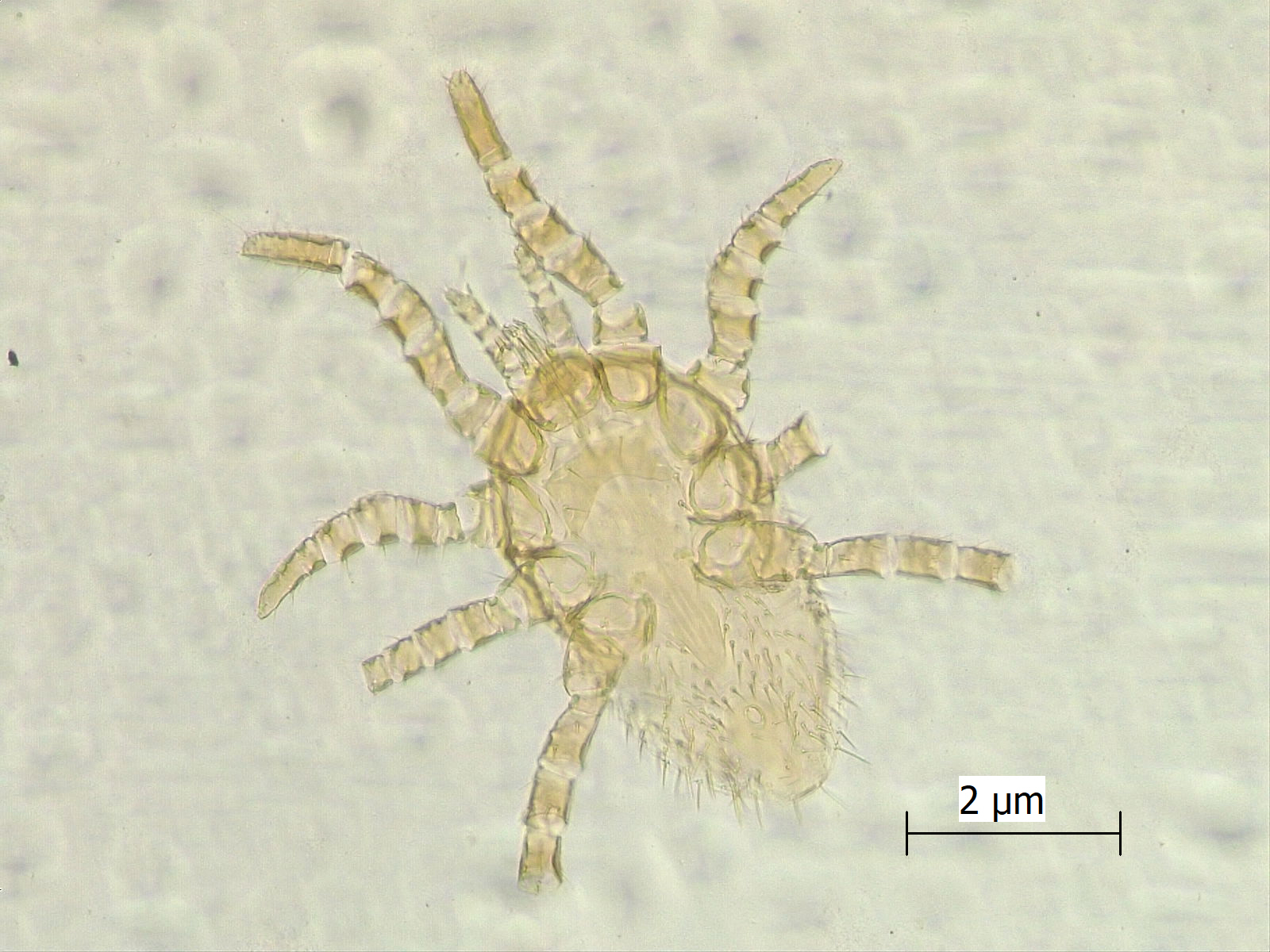


**SFig. 11:** Phylogenetic tree based on an alignment of ~400 bp of the 16S rRNA gene for *Ehrlichia* spp., using Maximum Likelihood method and K2+G as evolutionary model. Numbers at nodes correspond to the bootstrap (1000 replicates). The sequence obtained in the present work is highlighted in red. *Anaplasma marginale, Anaplasma phagocytophilum,* and *Rickettsia rickettsii* were used as outgroups.


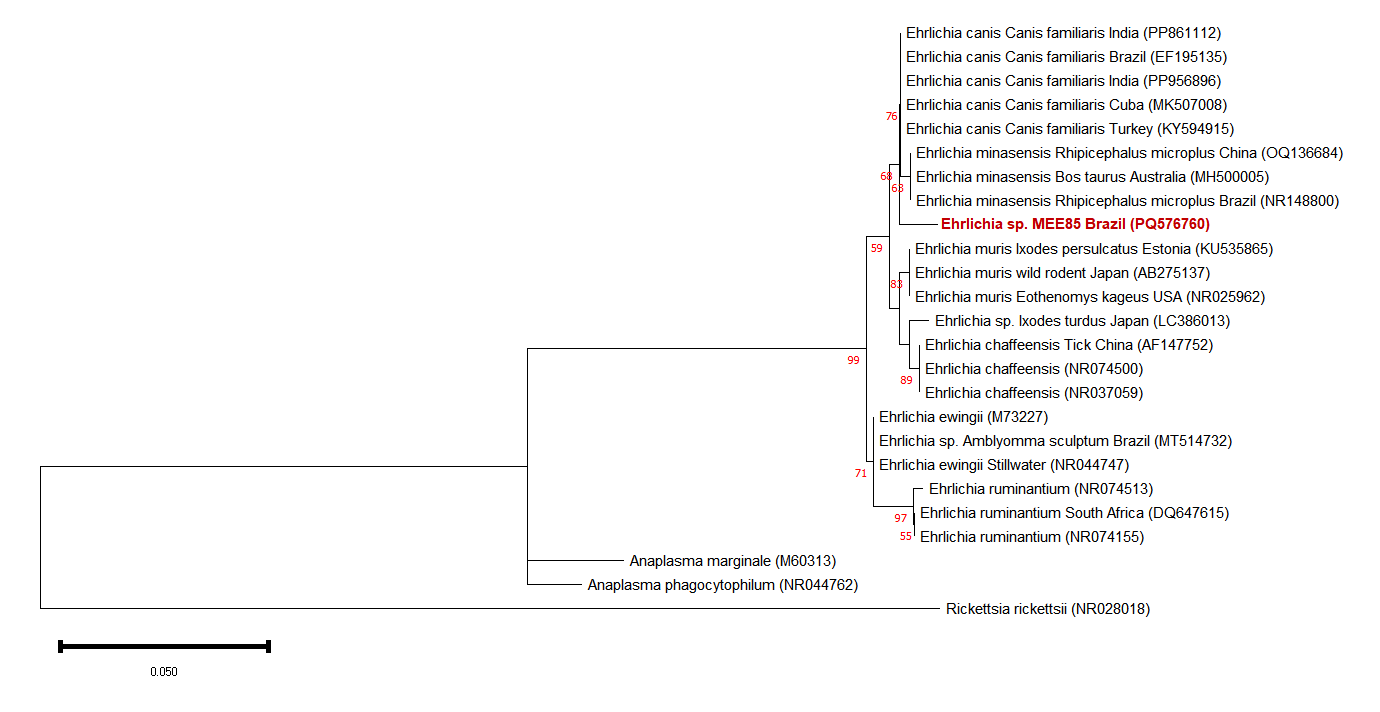


**SFig. 12:** Phylogenetic tree based on an alignment of ~600 bp of the 16S rRNA gene for hemoplasmas, using Maximum Likelihood method and TN93+G as evolutionary model. Numbers at nodes correspond to the bootstrap (1000 replicates). The sequences obtained in the present work are highlighted in red. *Mycoplasma pneumoniae* and *Bacillus subtilis* were used as outgroups.


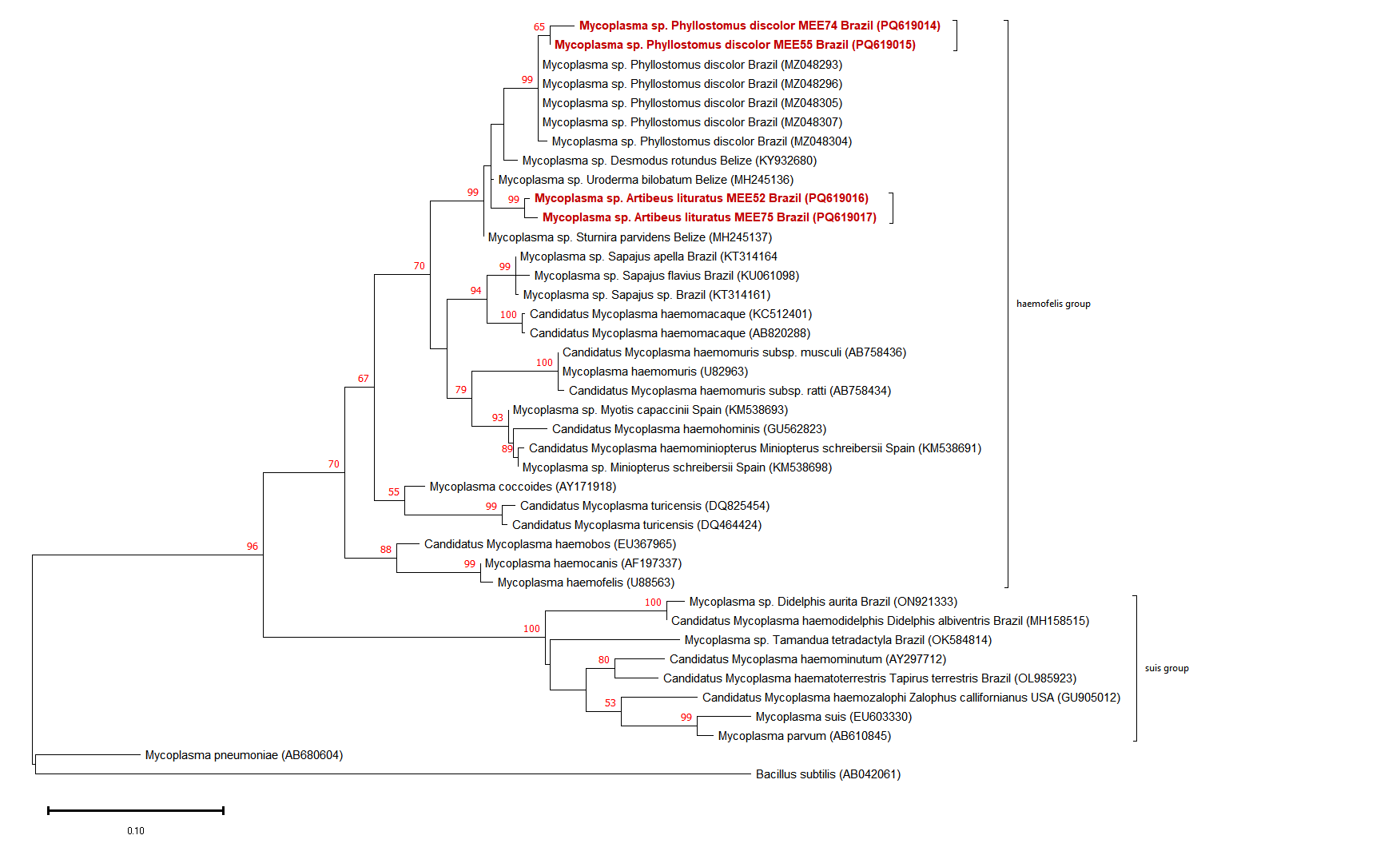


**SFig. 13:** Phylogenetic tree based on an alignment of ~610 bp of the 23S rRNA gene for hemoplasmas, using Maximum Likelihood method and T92+G+I as evolutionary model. Numbers at nodes correspond to the bootstrap (1000 replicates). The sequence obtained in the present work is highlighted in red. *Mycoplasma pneumoniae* was used as outgroup.


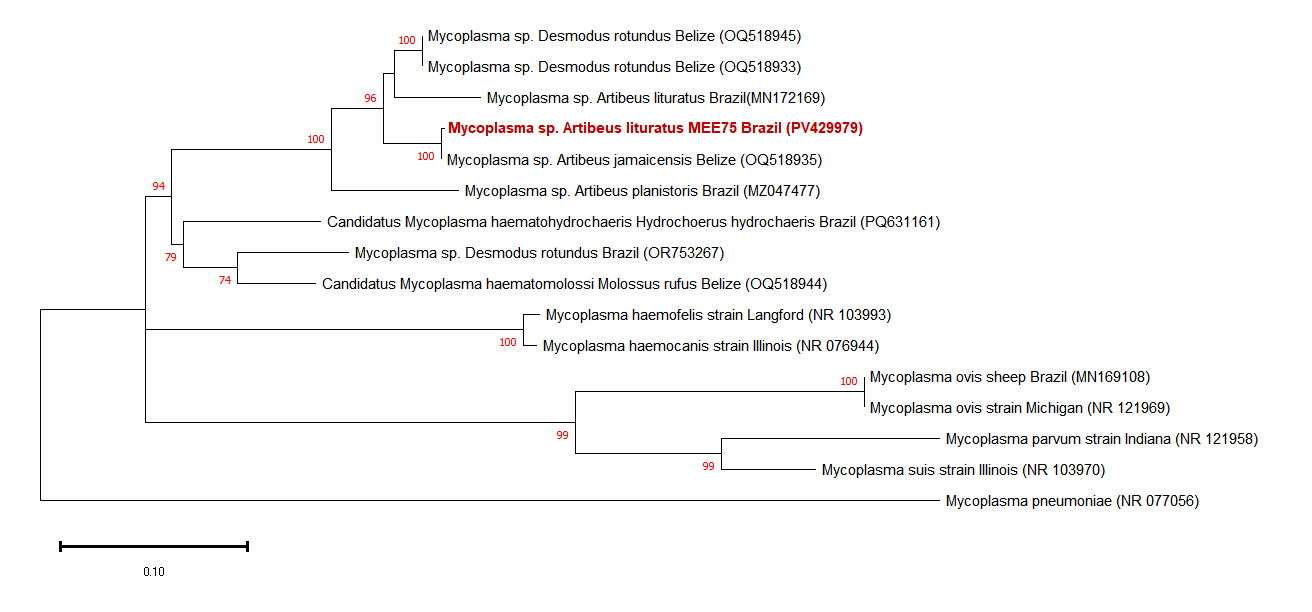

Supplement: Supplementary file 2 — Supplementary Material 2 [file 11259_2026_11341_MOESM2_ESM.docx]
